# The Prevalence of Vaping and Smoking as Modes of Delivery for Nicotine and Cannabis among Youth in Canada, England and the United States

**DOI:** 10.3390/ijerph16214111

**Published:** 2019-10-25

**Authors:** Fathima Fataar, David Hammond

**Affiliations:** School of Public Health & Health Systems, University of Waterloo, Waterloo, ON N2L 3G1, Canada; ffataar@uwaterloo.ca

**Keywords:** vaping, cannabis, nicotine, youth

## Abstract

*Background:* Vaping has become an increasingly common mode of administration for both nicotine and cannabis, with overlap among users, devices, as well as nicotine and cannabis companies. There is a need to understand patterns of use among youth, including the way nicotine and cannabis are administered. *Methods:* Data are from Wave 2 of the ITC Youth Tobacco and Vaping survey, an online survey conducted in 2018 among 16–19 year-olds recruited from commercial panels in Canada (*n* = 3757), England (*n* = 3819), and the U.S. (*n* = 3961). The prevalence of past 30-day vaping nicotine, non-nicotine and cannabis substances, as well as cannabis modes of use was examined. Logistic regression models examined between country differences in prevalence. *Results:* Past 30-day cannabis use was highest among Canadian youth (16.6%), followed by youth in the U.S. (13.8%) and England (9.0%). Vaping e-cigarettes was substantially more prevalent than vaping cannabis in all three countries. All forms of cannabis use were higher among Canadian and U.S. youth compared to England (*p* < 0.001 for all). Past 30-day cannabis users in the U.S. were more likely to report vaping cannabis oil (30.1%), and consuming solid concentrates such as wax and shatter (30.2%), compared to cannabis users in Canada (18.6% and 22.9%) and England (14.3% and 11.0%; *p* < 0.001 for all). *Conclusions:* Youth are administering cannabis and nicotine using a wide diversity of modes. Cannabis users in the U.S.—where an increasing number of states have legalized medical and non-medical cannabis—reported notably higher use of more potent cannabis products, including cannabis oils and extracts.

## 1. Introduction

In most Western countries, cigarette smoking remains the dominant form of nicotine use [1]. However, in recent years, the use of other devices for nicotine consumption, in particular vapourizers, have become increasingly common. Commercial vapourizers emerged in the late 1990s as an alternative mode of cannabis use. These vapourizers were typically large devices that heated dried cannabis herb below the point of combustion to the point of vapourization [2]. Beginning in 2003, smaller, portable vapourizers emerged as ‘electronic cigarettes’ and heated nicotine in a solution rather than from dried leaf, as was the case for the early cannabis vapourizers. More recently, vapourizers in the cannabis market have followed a similar transition, with greater use of cannabis extracts in oil ‘pods’ or vape pens, alongside devices that use dried herb [3,4,5]. This is expected to further expand as cannabis companies partner with tobacco companies to benefit from vaping technology [6]. The current paper uses the term ‘e-cigarette’ or ‘vaping’ to refer to nicotine and other non-cannabis forms of vaping while ‘cannabis vaping’ is used to refer to the cannabis-specific mode of administration.

E-cigarette use is most prevalent among youth and young adults in countries such as Canada, England, and the United States (U.S.). While the sale of e-cigarettes and e-liquids is illegal for minors to purchase in these countries, they are still widely accessed by youth [7]. Recreational cannabis is currently legal in 11 U.S. states and in Canada, as of October 2018. Medical cannabis is legal in 33 U.S. states and has been legal in Canada since 2001. The minimum legal age for e-cigarettes and cannabis is 18 or 19 in Canada (depending upon province of residence); in the U.S., the minimum legal age for e-cigarettes ranges from 18 to 21, and is 21 for cannabis in all states in which it is legal. E-cigarette use has increased over the past decade in all three countries, although to different extents [8,9,10]. Substantial increases in youth vaping were observed between 2017 and 2018 in Canada and the U.S., consistent with increases in JUUL, the leading brand of nicotine vaping devices in North America, and other nicotine salt ‘pod’ devices [7,10,11]. Over the same period, smoking tobacco has declined, although smoking rates may have stabilized more recently [8,10,12]. E-cigarettes can be used with or without nicotine-containing liquid or ‘juice’ [13]. A review looking at published studies of e-cigarette prevalence among youth between 2014 and 2016 found that most studies did not report whether nicotine or non-nicotine products were being used [14]. In Canada, it was reported that about 4% of Ontario high school students reported using an e-cigarette with nicotine, while 10% used non-nicotine liquids [15]. Data from a nationally representative sample of U.S. students found that among e-cigarette users, about 20% of 10 and 12th graders, and 13% of 8th graders used nicotine, while about 65% across all grades reported using non-nicotine products [16]. 

There is less published evidence on cannabis vaping among youth. Despite varying legal availability of cannabis products for adults in Canada, England and the U.S., the sale of cannabis to minors remains illegal in all three countries. While smoking cannabis remains the most common method of use among adults and youth, alternate modes of delivery, such as edibles and the use of e-cigarettes to vape cannabis products, are becoming increasingly popular [5]. A recent survey of adolescent cannabis users in the U.S. found that smoking was the most common mode of intake; however, almost half reported lifetime cannabis vaping [17]. Studies with U.S. college students have reported lifetime cannabis vaping prevalence ranging from 9% to 29%, while prevalence among high school students has been reported between 5% and 9% [18,19,20,21]. There are less data available on modes of cannabis use among youth in Canada and England. A study of students in Canada’s largest province, Ontario, reported that 8% of high school students aged 15–18 vaped cannabis [22]. Studies have consistently found a positive association between e-cigarette use and cannabis vaping, including among youth [18,19,20,21,23]. More research is needed to better understand not only what nicotine and cannabis products young people are using, but how they are consuming them.

One important methodological question is the extent to which population-based surveys are able to distinguish between vaping nicotine and cannabis. The language used to refer to “e-cigarette use” has evolved over time: ‘vaping’ is now widely used to refer to both ‘e-cigarettes’ and using a vapourizer for cannabis, alongside the emergence of new terms, such as “JUULing”. The terminology used to determine *what* is being vaped varies across national surveys. Questions range from general wording, such as “Have you tried an e-cigarette?” to much more specific items, such as “Have you ever used an e-cigarette to inhale another drug or substance other than nicotine?” [21,24]. While some questions differentiate between nicotine and non-nicotine substances, few explicitly establish whether non-nicotine substances include cannabis or other substances. Although the ability of youth to accurately self-report nicotine content is unclear, the different types of vaping and terminologies used represent a challenge to measuring the prevalence of different forms of nicotine and cannabis use [12,25,26].

While the prevalence of e-cigarette use and cannabis vaping has been examined within individual countries, there is little data available to be able to directly compare between country differences among youth. The current study captured data from youth in Canada, England and the U.S., providing the unique opportunity for comparisons between these countries, where the regulatory frameworks for nicotine and cannabis products differ. With the focus on youth in Canada, England and the U.S., the purpose of the study was: (1) To assess the prevalence of various modes of nicotine and cannabis use, with a focus on vaping and smoking. (2) To examine the extent of overlap between vaping and smoking both cannabis and nicotine products. (3) To examine differences in patterns of use between youth in Canada, England and the U.S. (4) To examine the proportion of youth who may be incorrectly classified as ‘e-cigarette’ users when they had only vaped cannabis.

## 2. Materials and Methods

### 2.1. Data source

Data are from Wave 2 of the International Tobacco Control Policy Evaluation Project (ITC) Youth Tobacco and Vaping Survey conducted in Canada, England, and the United States. Online surveys were conducted in August/September of 2018. Eligible respondents included youth aged 16 through 19 at the time of recruitment. Respondents were recruited through Nielsen Consumer Insights Global Panel and their partners’ panels, either directly or through their parents. The Nielsen panel was recruited using both probability and non-probability sampling methods in each country. Nielsen selected random samples from the online panel(s) in each country. Email invitations with a unique link were sent to a random sample of eligible panelists. The survey was conducted in English, as well as in French in Canada, and took approximately 15 minutes to complete. The same survey measures were used in all countries, with the exception of race/ethnicity, region and education questions, which were based on census questions in each country.

In the three countries, 10,980 Wave 1 respondents (81.5% of the 13,468 W1 respondents) were invited to participate in Wave 2 (*n* = 2726 in Canada; *n* = 4214 in England; *n* = 4040 in the U.S.). From these invitations, 1307 (11.9% of those invited; 10.1% of all Wave 1 respondents) surveys were completed and retained for analysis (n = 281 in Canada; *n* = 604 in England; *n* = 422 in the U.S.). However, since some invitations were at the parent level, the individual respondents may differ between waves—within the analytic sample, *n* = 1115 respondents were matched (using age and sex) as the same respondents as Wave 1. For the current study, only data from respondents who were not participants in Wave 1 were included in the analysis (*n* = 192).

In the three countries, a total of 512,035 invitations were sent to ‘new’ panelists (61,630 directly to youth, 450,405 to parents): *n* = 261,376 in Canada (30,660 youth and 230,716 parents), *n* = 125,998 in England (14,045 youth and 111,953 parents), and *n* = 124,661 in the U.S. (16,925 youth and 107,736 parents). Of these, 11,608 (2.3% of those invited) completed the survey and were retained for analysis. A full description of the survey and study methods can be found in the Technical Report [27]. 

This study received ethics clearance through the University of Waterloo Research Ethics Committee (ORE #21847), and the King’s College London Psychiatry, Nursing & Midwifery Research Ethics Subcommittee. All participants provided informed consent.

### 2.2. Measures

#### 2.2.1. Socio-Demographic Measures

Socio-demographic measures included age at time of survey, sex at birth (male/female), socioeconomic status, and race/ethnicity. Socioeconomic status was assessed using a question which asked about perceived family financial situation. To allow for cross-country comparisons, the country-specific race/ethnicity measure was recoded to ‘White/Caucasian only’ vs. ‘all others’.

#### 2.2.2. Vaping and Smoking Measures

Respondents were asked yes/no questions about having ever smoked cigarettes and using e-cigarettes/vaping (“Have you ever tried cigarette smoking, even one or two puffs?” and “Have you ever tried an e-cigarette/vaped, even one or two puffs?”). Respondents who answered yes were asked to indicate: “When was the last time you smoked a cigarette, even one or two puffs?” and/or “When was the last time you used and e-cigarette/vaped?” on the following scale: Earlier today/Not today but sometime in the past 7 days/Not in the past 7 days but sometime in the past 30 days/Not in the past 30 days but sometime in the past 6 months/Not in the past 6 months but sometime in the past 12 months/1 to 4 years ago/5 or more years ago. These questions were used to derive ever and past 30-day users.

Respondents who indicated that they used an e-cigarette/vaped in the past 30 days were also asked: “Do the e-cigarettes, cartridges, pods, or e-liquids that you currently use contain nicotine?” (Yes/No/I don’t know if they contained nicotine or not).

#### 2.2.3. Cannabis Measures

Cannabis use was assessed with the question: “When was the last time you used marijuana/cannabis?” (I have never used marijuana/cannabis/Earlier today/Not today but sometime in the past 7 days/Not in the past 7 days but sometime in the past 30 days/Not in the past 30 days but sometime in the past 6 months/ Not in the past 6 months but sometime in the past 12 months/1 to 4 years ago/5 or more years ago). These questions were used to derive ever and past 30-day users.

Respondents who indicated that they used cannabis in the past 30 days were asked about modes of use with the following yes/no questions: “In the last 30 days, did you: 1. Smoke marijuana/cannabis without tobacco 2. Smoke marijuana/cannabis with tobacco in a joint or blunt 3. Use a waterpipe/bong to smoke marijuana/cannabis 4. Use a vapourizer to heat dried marijuana/cannabis leaves or herb 5. Use an e-cigarette to vape marijuana/cannabis oil or liquid 6. Eat or drink marijuana/cannabis in a food or a beverage 7. Use marijuana/cannabis extracts, including oil, wax or shatter 8. Use another form of marijuana/cannabis.”

#### 2.2.4. Cannabis Vaper Misclassification Measures

To assess whether cannabis only vapers were being incorrectly categorized as e-cigarette users, respondents who reported ever using e-cigarettes/vaping and ever vaping cannabis were asked the following yes/no questions: “Just to confirm, have you ever: 1. Vaped e-liquids with nicotine and 2. Vaped other e-liquids without nicotine or marijuana/cannabis.” Similarly, those who reported ever using e-cigarettes/vaping and vaping cannabis in the past 30 days were asked: “Just to confirm, in the last 30 days have you: 1. Vaped e-liquids with nicotine and 2. Vaped other e-liquids without nicotine or marijuana/cannabis.”

### 2.3. Analysis

From the original sample of 11,800 respondents, 442 were excluded for missing data on e-cigarette or vaping outcomes, for a final sample size of 11,358 retained for analysis. Sample weights were constructed for each country based on age, sex, geographic region, race/ethnicity (U.S. only), and rescaled to the sample size in each country (see Technical Report for details) [27]. Logistic regression models were estimated to examine between country differences in modes of nicotine and cannabis use using an ‘indicator’ variable for country, adjusting for age, sex, race/ethnicity (‘White’ vs. other) and perceived family socioeconomic status. Parallel logistic regression models were run for each outcome: among all youth and among past 30-day cannabis users. Adjusted Odds Ratios (AORs) and weighted estimates are shown in all cases. Analyses were conducted using SAS 9.4 (Cary, USA: SAS Institute Inc.).

## 3. Results

### 3.1. Sample Characteristics

Table 1 shows the weighted sample characteristics for each country. Table 1 also shows the prevalence of smoking, vaping and cannabis use, as reported elsewhere [7]. 

### 3.2. Prevalence of Different Modes of Cannabis Use

Table 2 shows the prevalence of different modes of cannabis use among all youth, while Table 3 shows the prevalence of different modes of cannabis use among past 30-day cannabis users only. Overall, smoking cannabis was the most frequently reported mode of use: approximately 90% of past 30-day cannabis users reported smoking cannabis, with a past 30-day prevalence among all youth of 14.6% in Canada, 12.1% in the U.S. and 8.4% in England.

As shown in Table 2 and Table 3, youth in England were more likely to smoke cannabis with tobacco compared to youth in Canada and the U.S.

Overall, 1.7% of youth in England, and 4.7% and 5.1% of youth in Canada and the U.S. respectively, reported vaping any type of cannabis. The odds of vaping cannabis were more than 3 times higher among youth in Canada and the U.S. compared to England (see Table 2). Differences were also observed in the type of cannabis being vaped. Among past 30-day cannabis users in Canada and England, vaping dried cannabis and cannabis oil/liquid were equally common. In Canada, 19.0% of past 30-day users vaped dried herb while 18.6% vaped oil or liquid, compared to 12.3% and 14.3% (respectively) among youth in England. In contrast, among U.S. cannabis users, 20.9% reported vaping dried cannabis while 30.1% vaped oil/liquid. Overall, cannabis users in the U.S. were more likely to vape cannabis oil/liquid than youth in Canada and England, as shown in Table 3.

Cannabis users in Canada and the U.S. were also more likely to report ‘other’ modes of cannabis use compared to youth in England, including the use of waterpipes and bongs, edible cannabis products, and cannabis extracts including wax, shatter, and oil (see Table 3). Waterpipe/bong was the second most commonly reported mode of cannabis use, used by 51.7% in Canada, 47.4% in the U.S. and 22.4% in England. Edibles were less likely to be used by youth in England at 19.4%, compared to 26.6% in Canada and 29.4% in the U.S. The use of cannabis extracts was highest among cannabis users in the U.S. at 30.2%, compared to 22.9% in Canada and 11.0% in England.

### 3.3. Prevalence of Tobacco and Cannabis Smoking and Vaping

Table 4 shows the overlap in smoking and vaping cannabis and nicotine/tobacco. In Canada and the U.S., approximately 17% of youth reported vaping e-cigarettes and/or cannabis in the past 30 days, compared to 9.7% in England. While vaping e-cigarettes with and without nicotine was equally prevalent in England (approximately 4% for both), the prevalence of vaping e-cigarettes with nicotine was higher in Canada and the U.S. at about 8% and 10%, respectively, compared to vaping e-cigarettes without nicotine, which was 5.7% in Canada and 6.8% in the U.S. Among youth who reported vaping any e-cigarettes in the past 30 days, 20.0% in England, 15.5% in Canada and 11.3% in the U.S. indicated that they did not know whether the e-cigarette they used contained any nicotine.

Across all countries, less than 2% of youth in all countries reported vaping cannabis only, with slightly higher levels in Canada (1.7%) compared to England (0.9%). However, the proportion who vaped both e-cigarettes and cannabis was greater in Canada (3.0%) and the U.S. (3.9%) than in England (0.7%). Additionally, youth in the U.S. were more likely to vape both e-cigarettes and cannabis compared to youth in Canada.

Approximately 20% of youth in all countries reported smoking tobacco and/or cannabis. The prevalence of smoking tobacco only was highest in England (11.0%) compared to Canada (7.8%) and the U.S. (7.0%). In contrast, the prevalence of smoking cannabis only was lowest in England at 2.9% compared to 7.3% in both Canada and the U.S. Smoking both tobacco and cannabis with or without tobacco was more common among youth in Canada at 7.3% when compared to both England (5.5%), and the U.S. (4.9%). Additionally, youth in Canada were most likely to smoke tobacco and cannabis without tobacco (see Table 4 for all contrasts).

### 3.4. Misclassification of Cannabis Vapers as e-Cigarette Users

The extent to which cannabis only vapers were being incorrectly categorized as e-cigarette users was examined by identifying those who reported ever smoking an e-cigarette/vaping, but then later indicated that they were only vaping cannabis. Overall, 1.3% (*n* = 149) of ever, and 0.3% (*n* = 39) of past 30-day cannabis only vapers were misclassified as e-cigarette vapers based on the standard question wording. Among past 30-day users, misclassification was greatest in the U.S. (0.6%, *n* = 25), followed by Canada (0.3%, *n* = 11) and England (0.1%, *n* = 3). Adjusting for this difference, the adjusted prevalence of 30-day e-cigarette vaping would be 8.7% in England, 14.3% in Canada and 15.4% in the U.S.

## 4. Discussion

The study findings highlight the evolving vaping market and the overlap between modes of administration for nicotine and cannabis among young people. While the overall prevalence of e-cigarette and cannabis use was lower in England compared to Canada and the U.S., as reported elsewhere [7,28], different patterns of use were also observed among nicotine and cannabis users. Vaping nicotine was substantially more prevalent than smoking nicotine with respect to past 30-day use. While the prevalence of ‘ever’ e-cigarette use and monthly use has been increasing for several years, other studies suggest that more frequent use, including daily and weekly vaping, has overtaken smoking among U.S. youth, with similar trends in Canada [7,10]. Increases in vaping frequency may reflect the evolution in e-cigarette design, particularly with respect to the emergence of nicotine salt products with delivery of high concentrations of nicotine.

The current findings also add to the existing evidence base that many youth report vaping non-nicotine e-cigarettes. In England, similar proportions reported vaping e-cigarettes with and without nicotine; although more youth in Canada and the U.S. reported vaping nicotine e-cigarettes, non-nicotine e-cigarettes were still commonly reported. Overall, the proportion of non-nicotine e-cigarettes in the current study was somewhat lower than previous estimates, which may reflect greater uptake of nicotine e-cigarettes, particularly in the North American market [15,16]. The accuracy with which youth are able to report nicotine content, and wording of the questions are other possible reasons for the observed differences. The current study found that between 12% and 20% of e-cigarette users did not know whether the e-cigarette contained nicotine, similar to previous studies [16,26]. Thus, the use of nicotine-containing vaping products among youth may be substantially higher than self-reported estimates would indicate.

In contrast to nicotine, smoking remains the dominant form of cannabis use among youth in all countries. The substantially greater health risk of smoking tobacco compared to cannabis may serve as a greater incentive for consumers to switch to vaping nicotine [29]. Alternatively, the greater commercial development of nicotine e-cigarettes, including marketing and the prominence of flavoured products, may account for this change.

Among those that vaped cannabis, similar proportions vaped dried cannabis and cannabis oils in Canada and England, whereas vaping cannabis oils was more common among U.S. youth. This difference may reflect the legalization of non-medical cannabis in U.S. states, which is characterized by a shift towards more processed cannabis products, including cannabis oils and extracts [4,30]. Although the basic principles of vapourization are the same with respect to dried herb versus cannabis oils, the typical THC (delta-9-tetrahydrocannabinol) levels represent an important difference: the THC levels of commercially available dried herb are typically between 15% to 20%, compared to 70% for vaping oils. In addition, THC oils have been implicated in more than 1000 cases of severe pulmonary diseases and 18 deaths in the U.S., possibly due to toxic constituents or contaminants in the oils [31]. Therefore, while a shift from smoking to vaping is encouraged based on lower-risk cannabis use guidelines [32], there may be important distinctions between vaping dried cannabis herb and processed vape oils, which are associated with greater potency and potential risks due to manufacturing standards. Further research should examine potential differences in patterns of use between vaping dried herb versus oils, including consumer awareness of the differences in potency.

The findings also highlight the prevalence of other forms of cannabis use, including edibles, and the use of other extracts, and waterpipes and bongs. Waterpipes and bongs were most popular in Canada and the U.S., whereas the prevalence of cannabis edibles was more similar to vaping cannabis in each country. The use of high potency extracts was substantially more popular in Canada and the U.S., which also reflects the greater establishment of the medical and non-medical cannabis industry. The use of high potency extracts such as wax and shatter warrants particular attention given the potentially greater health risks associated with these products.

The findings highlight several other interesting differences between countries. In particular, cannabis users in Canada and the U.S. most commonly report smoking cannabis without tobacco; the opposite was observed in England, where smoking cannabis with tobacco is more than double that in Canada and the U.S. This difference reflects well established trends in cannabis use, in which mixed tobacco and cannabis is more common in Europe compared to North America [33,34]. This is potentially important with respect to concerns about the concurrent use of cannabis and tobacco, which may increase health risks or reduce the likelihood of successful cessation of either product [35]. 

Findings from the current study suggest that misclassification of cannabis-only vapers as e-cigarette users does not significantly alter the estimated prevalence, with misclassification resulting in a difference of less than 1% in each country. Concerns have been raised about whether the term vaping is being more broadly interpreted to refer not only to nicotine products or non-nicotine liquid, but also to cannabis. The current survey tool provided respondents with a brief overview of e-cigarettes, which included images, before the questions pertaining to e-cigarette use. It appears that respondents interpreted the questions in reference to e-cigarettes for nicotine or non-nicotine liquids, but not cannabis. It is unclear if these findings would hold if there is no preamble about what e-cigarettes/vaping were referring to in the context of the study. This distinction will be especially important as cannabis oils/liquids become more widely available as cannabis regulations change within jurisdictions. Survey measures should aim to provide context and clear questions to better assess what is intended by the term vaping.

## 5. Limitations

The current study is based on the self-report of youth, which can be subject to recall and social desirability bias. In Canada and England as well as most U.S. states, non-medical cannabis was illegal at the time of the survey, which may result in underreporting use. Moreover, as was noted with vaping nicotine products, youth may not have an accurate awareness and understanding of nicotine and its presence within products. A further limitation is the use of non-probability-based samples and the 2.3% completion rate among those invited from the commercial panel. However, to address this issue, the sample was weighted to align with established population benchmarks within each country and estimates of vaping and smoking are highly consistent with national benchmark studies [7]. Unfortunately, we are unaware of any national studies in any of the three countries that have provided estimates of various modes of cannabis use for comparison with those reported in the current study.

## 6. Conclusions

Overall, e-cigarettes have emerged as the most common mode of nicotine delivery among youth across Canada and the U.S., whereas smoking remains the dominant form of delivery for cannabis. However, there are increasing signs that legalization of medical and non-medical cannabis in North America are associated with greater adoption of cannabis extracts, including cannabis oil for vaping, as well as edibles and other smoked extracts. Collectively, these forms are typically far more potent with higher THC levels than dried herb. Further research is needed to monitor forms of cannabis use, particularly as a greater number of jurisdictions legalize cannabis, including Canada in October 2018.

## Figures and Tables

**Table 1:** Sample characteristics by country (weighted).

Characteristic	England *n* = 3819	Canada *n* = 3758	U.S. *n* = 3961
Age, mean (SD)	17.6 (1.05)	17.5 (1.08)	17.5 (1.07)
Sex			
Male	51.1 (1952)	51.2 (1925)	50.9 (2015)
Female	48.9 (1867)	48.8 (1833)	49.1 (1946)
Ethnicity			
White	77.6 (2966)	47.7 (1791)	73.6 (2915)
Other	22.4 (853)	52.3 (1967)	26.4 (1046)
Perceived SES			
Not meeting basic expenses	2.6 (99)	4.0 (151)	4.3 (171)
Just meeting basic expenses	20.5 (781)	22.1 (831)	25.1 (995)
Meeting needs with a little left over	36.6 (1399)	34.2 (1284)	35.8 (1419)
Living comfortably	37.9 (1445)	35.9 (1348)	32.1 (1271)
Don’t know	2.5 (95)	3.8 (144)	2.7 (105)
Cigarette Smoking			
Never	60.1 (2296)	63.7 (2392)	67.0 (2654)
Ever	39.9 (1523)	36.3 (1366)	33.0 (1307)
Past 30 days	16.5 (629)	15.1 (568)	12.0 (474)
Vaping (E-cigarette use)			
Never	67.3 (2571)	62.8 (2358)	66.5 (2633)
Ever	32.7 (1248)	37.3 (1400)	33.5 (1328)
Past 30 days	8.8 (335)	14.6 (549)	16.0 (635)
Cannabis			
Never	78.0 (2979)	67.6 (2540)	72.9 (2886)
Ever	22.0 (840)	32.4 (1218)	27.1 (1075)
Past 30 days	9.0 (342)	16.6 (625)	13.8 (546)

Data are % (*n*) or mean (SD).

**Table 2:** Prevalence of past 30-day cannabis modes of use among all youth and binary logistic regression analysis of cannabis modes of use by country (weighted) *.

Cannabis Mode of Use	Prevalence of Mode of Use among all Youth			Logistic Regression Analysis of Cannabis Use Mode by Country		
	England (*n* = 3819)	Canada (*n* = 3757)	U.S. (*n* = 3961)	England ** vs. Canada	England ** vs. U.S.	U.S. ** vs. Canada
Smoke any cannabis	8.4 (321)	14.6 (549)	12.1 (483)	2.14 (1.84–2.40)	1.74 (1.49–2.03)	1.23 (1.08–1.41)
				*p* < 0.0001	*p* < 0.0001	*p* = 0.0235
Without tobacco	4.8 (183)	13.2 (497)	11.6 (458)	3.64 (3.03–4.38)	3.10 (2.58–3.74)	1.17 (1.02–1.35)
				*p* < 0.0001	*p* < 0.0001	*p* = 0.0200
With tobacco	6.6 (253)	6.1 (228)	4.8 (188)	0.95 (0.79–1.15)	0.80 (0.66–0.98)	1.18 (0.97–1.44)
				*p* = 0.6105	*p* = 0.0291	*p* = 0.0956
Vape any cannabis	1.7 (63)	4.7 (175)	5.1 (203)	3.34 (2.48–4.50)	3.83 (2.86–5.14)	0.87 (0.71–1.08)
				*p* < 0.0001	*p* < 0.0001	*p* =0.1997
Dried cannabis	1.1 (42)	3.2 (119)	2.9 (114)	3.32 (2.31–4.77)	3.10 (2.15–4.45)	1.07 (0.82–1.39)
				*p* < 0.0001	*p* < 0.0001	*p* = 0.5981
Cannabis oil or liquid	1.3 (49)	3.1 (116)	4.2 (164)	3.07 (2.15–4.38)	4.37 (3.11–6.15)	0.70 (0.55–0.90)
				*p* < 0.0001	*p* < 0.0001	*p* = 0.0044
Waterpipe/Bong	2.0 (77)	8.6 (323)	6.5 (259)	6.18 (4.72–8.09)	4.55 (3.45–5.99)	1.35 (1.14–1.61)
				*p* < 0.0001	*p* < 0.0001	*p* = 0.0005
Eat or drink cannabis	1.7 (66)	4.4 (166)	4.1 (161)	2.97 (2.22–3.97)	2.63 (1.97–3.53)	1.12 (0.90–1.41)
				*p* < 0.0001	*p* < 0.0001	*p* = 0.2941
Cannabis extracts: Oil, wax, shatter	1.0 (37)	3.8 (143)	4.2 (165)	4.81 (3.28–7.06)	5.51 (3.77–8.04)	0.87 (0.69–1.11)
				*p* < 0.0001	*p* < 0.0001	*p* = 0.2651
Other	0.3 (11)	0.7 (25)	0.7 (29)	2.76 (1.35–5.64)	2.90 (1.43–5.89)	0.95 (0.56–1.62)
				*p* = 0.0055	*p* = 0.0033	*p* = 0.8548

* Adjusted for age, sex, race/ethnicity and socioeconomic status; ** Reference group; Data are % (*n*) or adjusted odds ratios (AOR) with 95% confidence intervals (CI) and associated *p*-values.

**Table 3:** Prevalence of past 30-day cannabis modes of use among past 30-day cannabis users and binary logistic regression analysis of cannabis modes of use by country (weighted) *.

Cannabis Mode of Use	Prevalence of Mode of Use among Past 30-day Cannabis Users			Logistic Regression Analysis of Cannabis Use Mode by Country		
	England (*n* = 342)	Canada (*n* = 625)	U.S. (*n* = 546)	England ** vs. Canada	England ** vs. U.S.	U.S. ** vs. Canada
Smoke any cannabis	93.8 (321)	88.0 (549)	88.3 (483)	0.72 (0.45–1.14)	0.75 (0.47–1.21)	0.95 (0.66–1.37)
				*p* = 0.1570	*p* = 0.2432	*p* = 0.7955
Without tobacco	53.3 (183)	78.7 (497)	82.0 (458)	4.14 (3.08–5.57)	5.45 (3.94–7.53)	0.76 (0.56–1.03)
				*p* < 0.0001	*p* < 0.0001	*p* = 0.0732
With tobacco	73.9 (253)	36.4 (228)	34.5 (188)	0.18 (0.14–0.25)	0.19 (0.14–0.26)	0.97 (0.76–1.23)
				*p* < 0.0001	*p* < 0.0001	*p* = 0.7772
Vape any cannabis	18.4 (63)	28.0 (175)	37.1 (203)	1.83 (1.32–2.55)	2.95 (2.11–4.12)	0.62 (0.48–0.70)
				*p* = 0.0003	*p* < 0.0001	*p* = 0.0002
Dried cannabis	12.3 (42)	19.0 (119)	20.9 (114)	1.75 (1.19–2.57)	2.07 (1.39–3.07)	0.84 (0.63–1.13)
				*p* = 0.0045	*p* = 0.0003	*p* = 0.2598
Cannabis oil or liquid	14.3 (49)	18.6 (116)	30.1 (164)	1.62 (1.11–2.37)	3.27 (2.24–4.75)	0.50 (0.38–0.65)
				*p* = 0.0130	*p* < 0.0001	*p* < 0.0001
Waterpipe/Bong	22.4 (77)	51.7 (323)	47.4 (259)	4.87 (3.55–6.68)	4.15 (3.00–5.74)	1.17 (0.93–1.48)
				*p* < 0.0001	*p* < 0.0001	*p* = 0.0002
Eat or drink cannabis	19.4 (66)	26.6 (166)	29.4 (161)	2.69 (1.79–4.05)	4.27 (2.83–6.43)	0.89 (0.69–1.16)
				*p* < 0.0001	*p* < 0.0001	*p* = 0.4007
Cannabis extracts: Oil, wax, shatter	11.0 (37)	22.9 (143)	30.2 (165)	2.69 (1.79–4.05)	4.27 (2.83–6.43)	0.63 (0.48–0.83)
				*p* < 0.0001	*p* < 0.0001	*p* = 0.0009
Other	3.1 (11)	4.1 (25)	5.3 (29)	1.32 (0.63–2.73)	1.76 (0.85–3.64)	0.75 (0.43–1.30)
				*p* = 0.4624	*p* = 0.1304	*p* = 0.3022

* Adjusted for age, sex, race/ethnicity and socioeconomic status; ** Reference group; Data are % (*n*) or adjusted odds ratios (AOR) with 95% confidence intervals (CI) and associated *p*-values.

**Table 4:** Prevalence of past 30-day tobacco and cannabis use among all youth and binary logistic regression for outcome variables by country (weighted) *.

	England (*n* = 3819)	Canada (*n* = 3758)	U.S. (*n* = 3961)	England ** vs. Canada	England ** vs. U.S.	U.S. ** vs. Canada
Vaping						
Vape e-cigarette and/or cannabis	9.7 (370)	16.3 (612)	17.3 (684)	2.19 (1.89–2.53)	2.28 (1.97–2.63)	0.96 (0.85–1.09)
				*p* < 0.0001	*p* < 0.0001	*p* = 0.5434
Vape any e-cigarette	8.8 (335)	14.6 (549)	16.0 (635)	2.14(1.84–2.49)	2.28 (1.97–2.65)	0.94 (0.82–1.07)
				*p* < 0.0001	*p* < 0.0001	*p* = 0.3218
Vape with nicotine ^1^	4.1 (156)	8.3 (314)	9.9 (392)	2.78 (2.26–3.42)	3.17 (2.59–3.89)	0.88 (0.75–1.03)
				*p* < 0.0001	*p* < 0.0001	*p* = 0.1046
Vape without nicotine	4.0 (153)	5.7 (214)	6.8 (269)			
Vape with unknown nicotine content	1.7 (67)	2.3 (85)	1.8 (72)			
Vape e-cigarettes only	8.0 (307)	11.6 (437)	12.2 (481)	1.80 (1.53–2.12)	1.78 (1.51–2.09)	1.01 (0.88–1.17)
				*p* < 0.0001	*p* < 0.0001	*p* = 0.8797
Vape cannabis only	0.9 (35)	1.7 (63)	1.2 (49)	2.17 (1.41–3.35)	1.79 (1.14–2.80)	1.21 (0.84–1.74)
				*p* = 0.0005	*p* = 0.0105	*p* = 0.3011
Vape both e-cigarette & cannabis	0.7 (28)	3.0 (112)	3.9 (154)	4.46 (2.96–6.73)	5.94 (3.98–8.86)	0.75 (0.58–0.97)
				*p* < 0.0001	*p* < 0.0001	*p* = 0.0256
Smoking						
Smoke tobacco and/or cannabis	19.4 (740)	22.4 (843)	19.2 (762)	1.32 (1.17–1.48)	1.03 (0.92–1.16)	1.28 (1.14–1.43)
				*p* < 0.0001	*p* = 0.6205	*p* < 0.0001
Smoke tobacco only	11.0 (420)	7.8 (293)	7.0 (279)	0.69 (0.59–0.81)	0.55 (0.46–0.65)	1.26 (1.05–1.51)
				*p* < 0.0001	*p* < 0.0001	*p* = 0.0111
Smoke cannabis only	2.9 (111)	7.3 (275)	7.3 (288)	3.10 (2.46–3.90)	3.20 (2.55–4.03)	0.97 (0.82–1.15)
				*p* < 0.0001	*p* < 0.0001	*p* = 0.7131
Smoke both tobacco & cannabis with or without tobacco	5.5 (209)	7.3 (275)	4.9 (195)	1.48 (1.22–1.79)	0.92 (1.22–1.79)	1.60 (1.31–1.94)
				*p* < 0.0001	*p* = 0.4610	*p* < 0.0001
Smoke tobacco & cannabis without tobacco	3.0 (116)	6.2 (234)	4.8 (189)	2.40 (1.89–3.05)	1.72 (1.34–2.20)	1.40 (1.14–1.72)
				*p* < 0.0001	*p* < 0.0001	*p* = 0.0013
Smoke tobacco & cannabis with tobacco	4.7 (181)	4.4 (165)	2.5 (97)	0.93 (0.75–1.16)	0.52 (0.41–0.68)	1.78 (1.38–2.30)
				*p* = 0.5385	*p* < 0.0001	*p* < 0.0001

* Adjusted for age, sex, race/ethnicity and socioeconomic status; ** Reference group; Data are % (*n*) or adjusted odds ratios (AOR) with 95% confidence intervals (CI) and associated p-values; ^1^ Binary logistic regression for vaping with nicotine (vs without nicotine/unknown nicotine content).
